# Supplementary material for: Healthy or Not Healthy? A Mixed-Methods Approach to Evaluate Front-of-Pack Nutrition Labels as a Tool to Guide Consumers
Source: Nutrients. 2022 Jul 8;14(14):2801. doi: 10.3390/nu14142801 (PMC9318739; doi:10.3390/nu14142801)
Supplement: Supplementary file 1 [file nutrients-14-02801-s001.zip › nutrients-1783831-supplementary.pdf]

Table S1. Educational background and occupation of professional participants

Respondent	Age	Highest education	Occupation	Conflict of interest
PR1	42	HND	Business Owner (Inform. Techn.)	NA
PR2	37	Bachelor	Marketing Consultant	NA
PR3	29	Masters	Researcher - Ethics	NA
PR4	56	BEd Hons	School Principal	NA
PR5	31	BEd Hons	Technology Teacher	NA
PR6	55	HND	Consumer Journalist	NA
PR7	53	HND	Financial Advisor	NA
PR8	50	Masters	Futurist	NA
PR9	55	PhD	Economist	NA
PR10	49	PhD	Political Economist	NA
PR11	36	PhD	Social Anthropologist	NA
PR12	57	PhD	Researcher - Horticulture	NA
PR13	27	Bachelor	Small Business Owner (Traditional use products)	F
PR14	46	PhD	Business Owner (Food Ingredients)	F
PR15	40	PhD	Food Scientist	F
PR16	58	HND	Director (Nutraceutical Company)	F
PR17	66	Masters	Director (Food Labelling Consulting)	F
PR18	35	Hons	Farmer (Tea Production)	F
PR19	65	MBChB	Director (Food Analysis Consulting)	F
PR20	51	BSc	Food Scientist	F
PR21	43	MSc	Multinational Research and Development Executive	F
PR22	36	BSc	Innovation Manager	F
PR23	52	BSc Eng; BCom LLB	Attorney	F
PR24	54	PhD	Researcher - Agriculture and Food	F
PR25	53	Bachelor	Research and Policy Coordinator	F
PR26	61	PhD	Nutrition Consultant	HC & F
PR27	53	HND	Nurse	HC
PR28	44	BSc Hons	Dietician	HC
PR29	33	MBChB	Doctor	HC
PR30	36	Masters	Researcher - Epidemiology	HC
PR31	53	Masters	Public Health Consultant	HC
PR32	36	PhD	Researcher - Non-Communicable Diseases	HC
PR33	30	BSc Hons	Dietician	HC
PR34	45	MBChB	Chief Healthcare Officer	HC
PR35	60	MBChB	Consultant to Department of Health	HC
PR36	39	Masters	Nutrition Consultant	HC
PR37	40	PhD	Lecturer - Nutrition	HC

HC: Healthcare and related industries; F: Food Industry; NA: Not related to food or healthcare industries

Table S2. Demographic details of interview participants (consumers)

Participant	Age	Gender	Occupation	Relationship status	Children
CN1	49	Female	Business Developer	In a relationship	0
CN2	43	Male	Project Manager	Single	0
CN3	32	Female	Engineer	Married	0
CN4	41	Female	Entrepreneur	Married	2
CN5	47	Male	Lecturer	Married	2
CN6	45	Female	Domestic Worker	Widowed	2
CN7	35	Male	Continuous Improvement	Single	2
CN8	72	Female	Former Librarian	Divorced	0
CN9	34	Female	Technician	Married	2
CN10	29	Female	Technician	Single	0
CN11	36	Female	Marketing Officer	Married	2
CN12	45	Female	Housewife	Married	3

Table S3. Label improvement themes from qualitative interviews

Theme	Recommendation	Illustrative quotes	Participant	COI
**Make it clearer**	Increase font size	*Something that can be easily seen. You don't stop to try and look with a magnifying glass at the fine print.*	PR32	HC
		I think your major ingredients, the ingredients that everybody is knowledgeable on, should be bigger, it should be more visible… I think people are not reading it because they can't see it.	PR27	HC
		We try to put so much on this pack that the font size is pathetic… We haven't gotten to that point where we actually try and help people read what's on the pack… I don't think we've been that discerning here yet. I think that a lot of the information is fairly illegible, or too small for people to actually read.	PR21	F
		They are printed so bloody small that I don't know anybody that can actually read them.	CN1	C
		By law, it should be - so the larger the pack size, you know, the requirement of the one millimeter, but when you have more space, there's a proportionality towards letter sizes and so on.	P36	HC
		So here's an example. This is a product my parents brought over [from the US]. So this is black on white. I think you can do white on black on white... And that's the standard.	PR2	NA
	Legibility (e.g. poor contrast)	*Legibility criteria! At the moment the criteria simply say it must be legible, but the criterion is written in such a way that it's open to debate. Many people struggle to read the fine print on labels. Sometimes manufacturers do it completely innocently. They've got a graphic designer who's trying to make a beautiful picture and then they print with orange on a yellow background and crazy things like that.*	PR23	F
		Have you ever tried to read the back of the label of a bar of chocolate? It's written on navy blue, or dark red paper - in black writing… I can't read it, so what was the use of putting this on the label?	PR35	HC
	Plain language	*I don't want to know it's got sodium or sodium chloride or NaCl. I want to know: has this got salt or no salt?*	PR35	HC
		I think language… to be a little bit more conscious of how consumers actually read and interpret things … I just think that we could do a better job on the language we use when we put stuff on pack.	PR21	F
		I don't think the general public glances at it. They don't understand what it means. I mean, there's lots of big words there… It's got 809 milligrams of sodium. Is that too much? Is that too little? I mean, what do they take out of it?	PR29	HC
		Some ingredients I don't understand. It can be something like a certain oil, but then they'll use a science term for it... To a certain extent, I am consuming things that I honestly do not know.	CN10	C
**Make it simpler**	General (make it simpler)	*My view is trying to make the labelling simpler, eye catching from a simple standpoint… the sugar content is higher… It's about reducing the noise.*	CN12	C
		Simplification of labelling. With the new front-of-pack information that companies are starting to come up with, it helps to balance the fact that the information on the back of the label is so complicated, because certain key information is provided right in front… I wouldn't use colors. I would just do it in black and white. Sort of like a picture…	PR23	F

Percentages (GDA type)	*The key nutrient thing on the front… It's got the amount of grams and it's on the front of the pack, which is quite clearly visible, and it stands out.*	PR32	HC
	Sugar would be a good one to start with because of the health problem that it presents in South Africa... It would be great if there was a big sign on every product that said, what percentage or how much sugar is in each one.	PR25	NA
	Give it in grams and then give the percentage of what you are supposed to have in a day.	CN10	C
Ratings or scales	*You can't just look at a food based on one specific nutrient. This is where governments bring in tools that help people make a decision in terms of an overall product rating, like the Health Stars in Australia… There's a move globally to interpretive models, because people don't have the background to make the decision based on one nutrient.*	PR37	HC
	Some kind of a number… I go buy coffee and it tells me on a scale of one to five how strong the coffee is. That's enough for most people… 1 to 5.	PR35	HC
Ratings or scales combined with colors	*So, if you had like a graded scale bar that shows you that you're already in the red, caution… A scale where there's a red and a green and then this sits in the green area. This is a good one and this is not so good, and then this is the reason why.*	PR32	HC
	I'd like a rated product: ingredients kind of in the red zone, those ingredients are yellow zone and these ingredients are totally safe. There's nothing you should worry about; you can give it to your child… I don't think the average person has nutritional knowledge… so if it's color coded… that would be helpful and quicker to assess.	CN3	C
Traffic light	*The traffic light system helps you. You don't have to go and look at each nutritional table separately. You can look at the lights and know… People are not going to look at a nutritional table but they're going to know "if I have a green, it's okay to give it to my kids; it's got red; I need to be cautious".*	PR15	F
Endorsement logo	*Something that can be easily seen… like the black Heart Foundation symbol, that's easily spotable… They won't even need to read the nutritional label. Something that can give people an instant 'that's a good thing, that's not a good thing'.*	PR32	HC
	Nobody wants to spend hours reading labels before they put it into the basket. They just want to see it's a green, or Heart Foundation… Tells the consumer quickly 'put this in your basket', or maybe if its red, maybe you should look at the label and see why they think it's red.	CN3	C
	My feeling is that we must try and simplify messages. I think something like a national health endorsement logo, however that would work, would actually be very powerful.	PR37	HC

	Warnings	*Like you put on the cigarette box – 'this product is dangerous to your health'. That is useful labelling. 'Sugar content is extremely high'.*	PR35	HC
		I think these front-of-pack warnings are really good because they need to have nutrient profiling models that look at not only the good nutrients and the bad nutrients, but look at them in conjunction and in ratio – and these are important to be done on an evidence base.	PR31	HC
		I think it's a good thing if certain items that in food must be listed on the front as: 'BEWARE the following are in here'. I wouldn't use the word 'beware' but I think we're heading towards where they have to say prominently 'SUGAR"…	CN2	C
		It would be good if on the things that are unhealthy, things that might cause someone's health to deteriorate, to be written in red or highlight it or in bold or something like that, just to create that awareness….	CN7	C
		If eating too much of that food, let's say polony, you keep on eating it every day as your staple food. They need to disclose that continuously eating this product, or they must have like a 'you shouldn't eat more than this amount of this food' or 'you cannot eat it more than five days in a week'… Everyone can understand it and then they can make an informed decision. I can eat it, but let's just not eat it every single day. So that would be fair or responsible labelling.	CN9	C
	Health claims	*If you read through the function claims, for instance… Some of it would be quite useful to have and to include on products… It will be so much easier to educate an already well-educated consumer.*	PR37	HC
		What is in the product that makes it beneficial, what is the aspect of health that it is beneficial to and what is the dosage.	PR33	HC
		I think health claims are good. I think it needs to be there on the appropriate products in the appropriate way. The big question is just: what are the appropriate products? And what is the appropriate way?	PR30	HC
		It would be wonderful to see the health claims come through. Good claims… substantiated claims can be made. That does make it a little bit easier for consumers to be able to decide between the products because the benefits are highlighted.	PR28	HC
		If we were able to make claims on food labels, to say: "these carrots contain beta carotene, which is good for the eyesight", it would lead to a healthier society because people will say, 'Okay, I need to buy carrots because this is what it's going to do for me'.	PR13	F
	Teaspoons	*The teaspoon thing is something that could make a difference for especially the widely, commonly used products, for sure.*	PR29	HC
		The other thing that I think is valuable is [Retailer name], on their muesli, they say 'per serving this now only contains one and a half teaspoons of sugar'… Everyone understands what a teaspoon of sugar looks like and I realized in the hospital that it also works very well.	PR30	HC
		I use teaspoons. Coke contains 115 grams of sugar. That's 23 teaspoons. Immediately I've got a reference point. I can visualize the two that I'm putting in and then times that by 10.	PR16	F
**Make it smarter**	Technology to decode nutrition	*Technology, I think, will play a very big role. Even in rural areas, people have mobile phones. At the very least, they have WhatsApp. Even if it's not an app. Look at what the Health Department has done now with Covid: they've got that number that you add and then they send you the latest stats… I think it's a brilliant initiative from them. We could have something like that.*	PR32	HC

	Probably, we could get a long way if we actually create more pictures, so that it's easier to understand. Or these days, using smartphones… You can actually create an app that will interpret the label for you. Or gamification, so it actually just becomes more fun, because it's a boring read.	CN1	C
	We could put a little digital thing, so I could scan it… if it could be somehow barcoded in and I could download an app and it could just [say] *'if you have five of these you don't need anything else for the day'.*	CN4	C
	Like a calorie tracker, but what that app does, you can actually go to that barcode and then it can actually pick up the food... It can just be a small pop up page about that certain food, just to explain it better.	CN10	C
	Everyone has got a phone. So with the QR code, you can quickly scan, and you can integrate that into the database that either forms a diet point of view or why this product is good for you, or the concerns… I do believe that, in future, will come. It's a matter of time.	PR14	F
	I think in terms of making it easier to consume the data, you could do a barcode or QR code that goes up [to a database] and gives you more detailed information or even points to some sort of educational content… the more convenient way of doing it would be somehow having a collection of items scanned all at once – so that you could open the fridge and the fridge could give you a score: 'your fridge health is seven'. That is the only way I can see it being convenient, because nobody's going to continually scan all this stuff in the fridge, unless the fridge did it.	PR1	NA
	In 10 years from now, I wouldn't be surprised if you're barcode scanning. You'll scan the item and it goes 'you've got a lot of sugar in what you bought today'.	CN2	C
	I think a way to make labels more personalized… Labels that are much more relevant to you. Maybe through technology you could actually understand your profile and how this product fits into your needs. … You have some sort of interface that's through technology that makes it more personal for you, and relevant to you.	E30	F

COI: Conflict of interest; HC: Healthcare and related industries; F: Food Industry; NA: Not related to food or healthcare industries

Table S4. Challenges related to food labelling as identified by interview participants

Challenge	Illustrative quotes	Participant	COI
Education	*I don't think that now you must only put pictures and make it visual so that someone knows that red is bad. It's a nanny kind of state thing to do. I think that you'll never progress, because a food label is only one way of helping someone make decisions. If you make that oversimplified, then how are they going to get the next concept of how this translates into my health and how I can enjoy things in moderation. It's just a stepping stone… simplify it if you can, but teach people.*	PR36	HC
	A start, a good start would be that people just had nutritional education and they are reminded about the basics – eating fruits and vegetables and basic nutrition education. Thereafter, the next layer will be, 'what do the different ingredients mean?', 'how do you read a nutritional table?'	PR28	HC
	You're not educating the public of making an informed decision at the end of the day. I don't think it's changing much more. That's not fixing the problem.	PR29	HC
	I've got an education, but I don't necessarily know all the ingredients. I didn't do Food Science… I don't think people have that in-depth knowledge of food that you would think the average educated person has.	CN3	C
	Let's say, on a sweet or something, it would be written 'no colorants used'. Do people really understand what that means? It goes back to education and being aware, about what is the written on that package and what people should expect... I don't even know; I think most people know what to expect in a certain product.	CN7	C
	To me, what is lacking, is the education. How do you actually use that information in a practical, palatable way…? A product that he or she is consuming in terms of nutritional makeup, how does that fit into the greater diet? Or what the consumer wants to achieve from a nutritional status. I think from an education point of view, there is definitely still scope for improvement.	PR14	F
	I genuinely do believe that the consumer needs to be part of the educational exercise – be made aware of what a normal value is, what's an elevated value etc.	PR16	F
Differing needs	*We're starting to understand that the microbiome is far more important than what we ever anticipated… GI has been heavily influenced by the microbiome and now we're starting to understand why the results are always all over the show. The evidence is that the microbiome metabolizes carbohydrates differently according to your microbiome, and so two people [will be] eating the same product, but one will have a high GI and the other one will have a low because of their [differing] microbiome structures, or makeup.*	PR19	HC/F
	Putting it on the front of pack, you're telling someone in short, these are the five nutrients that you should be concerned about. But because everyone's needs are different.	PR36	HC
	The biggest problem, I think, is that people need to put it into perspective. What does it mean for me?... What's the benefit?...	PR34	HC
	I think that can be a bit deceptive. The average person needs 8400 kilojoules, but for some people that is way over… because different people have different requirements. It's very difficult to on a population level to say the average person [needs] 8400 kilojoules and therefore we're working on a one size fits all.	PR30	HC

COI: Conflict of interest; HC: Healthcare and related industries; F: Food Industry; NA: Not related to food or healthcare industries

Table S5. Cronbach alpha for HCS

HCS Survey Question	Variable item	Total correlation	Alpha if deleted
I reflect about my health a lot	Health Reflection	0.66	0.93
I'm very self-conscious about my health	Health Conscious	0.72	0.92
I'm generally attentive to my inner feelings about my health	Health In Feel	0.73	0.92
I'm constantly examining my health	Health Examine	0.77	0.92
I'm alert to changes in my health	Health Change Alert	0.75	0.92
I'm usually aware of my health	Health Aware	0.78	0.92
I'm aware of the state of my health as I go through the day	Health State	0.78	0.92
I notice how I feel physically as I go through the day	Physical Feel	0.73	0.92
I'm very involved with my health	Health Involved	0.77	0.92

Cronbach's alpha and 95% confidence interval: 0.93 (0.92, 0.94); Standardized alpha: 0.93; Average inter-item correlation: 0.60

Table S6. Cronbach alpha for FOP label rating

Survey Question	Variable item	Total correlation	Alpha if deleted
On a scale of 1 to 10, rate how healthy you think this product is	Healthy	0.92	0.96
On a scale of 1 to 10, rate how good you think this product is for you	Good individual	0.93	0.95
On a scale of 1 to 10, rate how good you think this product is for your family	Good family	0.93	0.95
On a scale of 1 to 10, rate how likely it is that health conscious people will use this product	Healthy use	0.89	0.97

Cronbach’s alpha and 95% confidence interval: 0.97 (0.97, 0.97); Standardized alpha: 0.97; Average inter-item correlation: 0.89

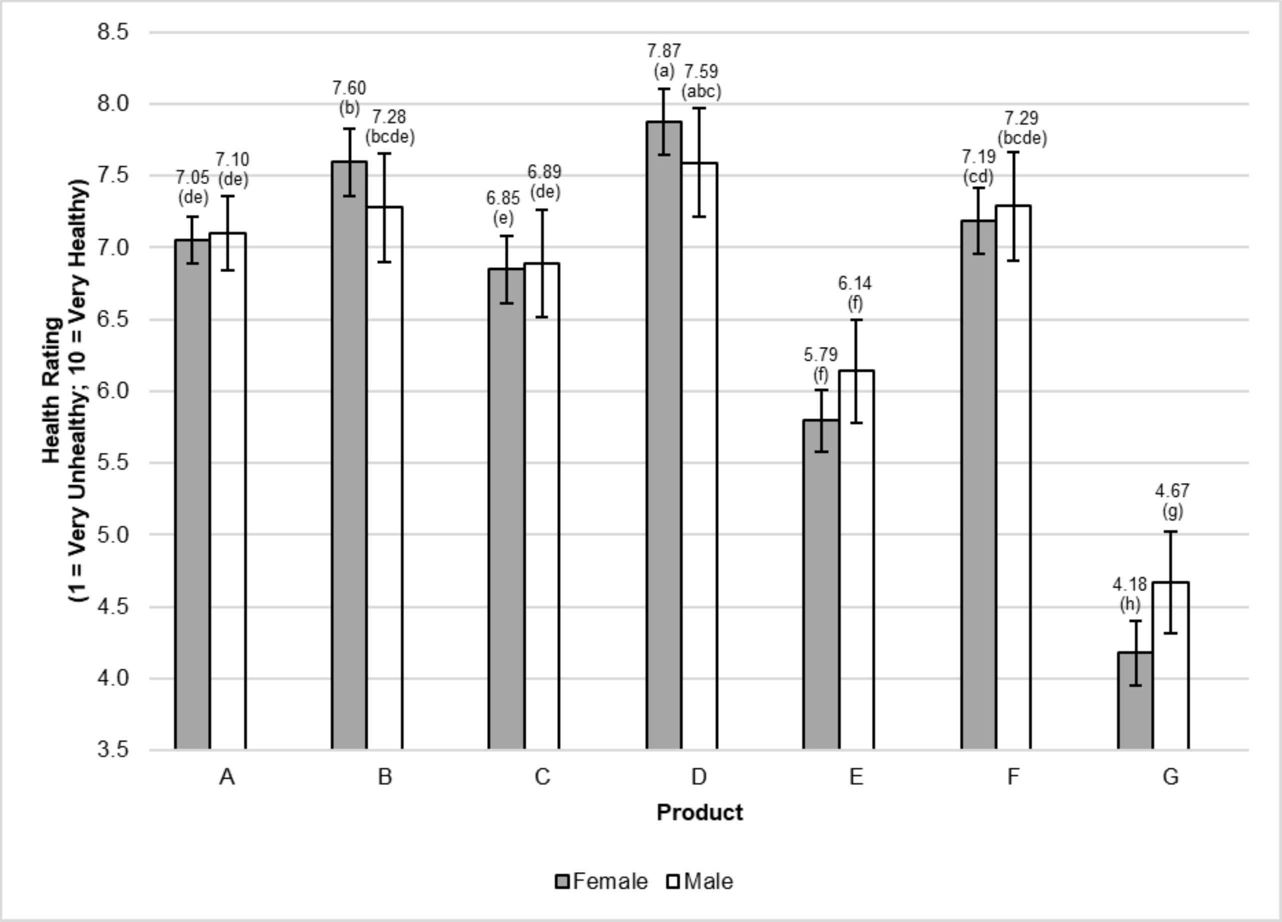

Figure S1. Gender-specific label ratings for fictitious cereal product with different FOP labelling. *Product A: control; Product B: high Health Star Rating; Product C: Guideline Daily Amount with a 'less healthy' nutritional profile; Product D: endorsement logo/low Glycemic Index claim; Product E: low Health Star Rating; Product F: Guideline Daily Amount with a 'healthy' nutritional profile; Product G: warning. Differences with a significance level of 5% ($p < 0.05$) were considered statistically significant and are indicated by different alphabetical letters on the graph (a-f).*

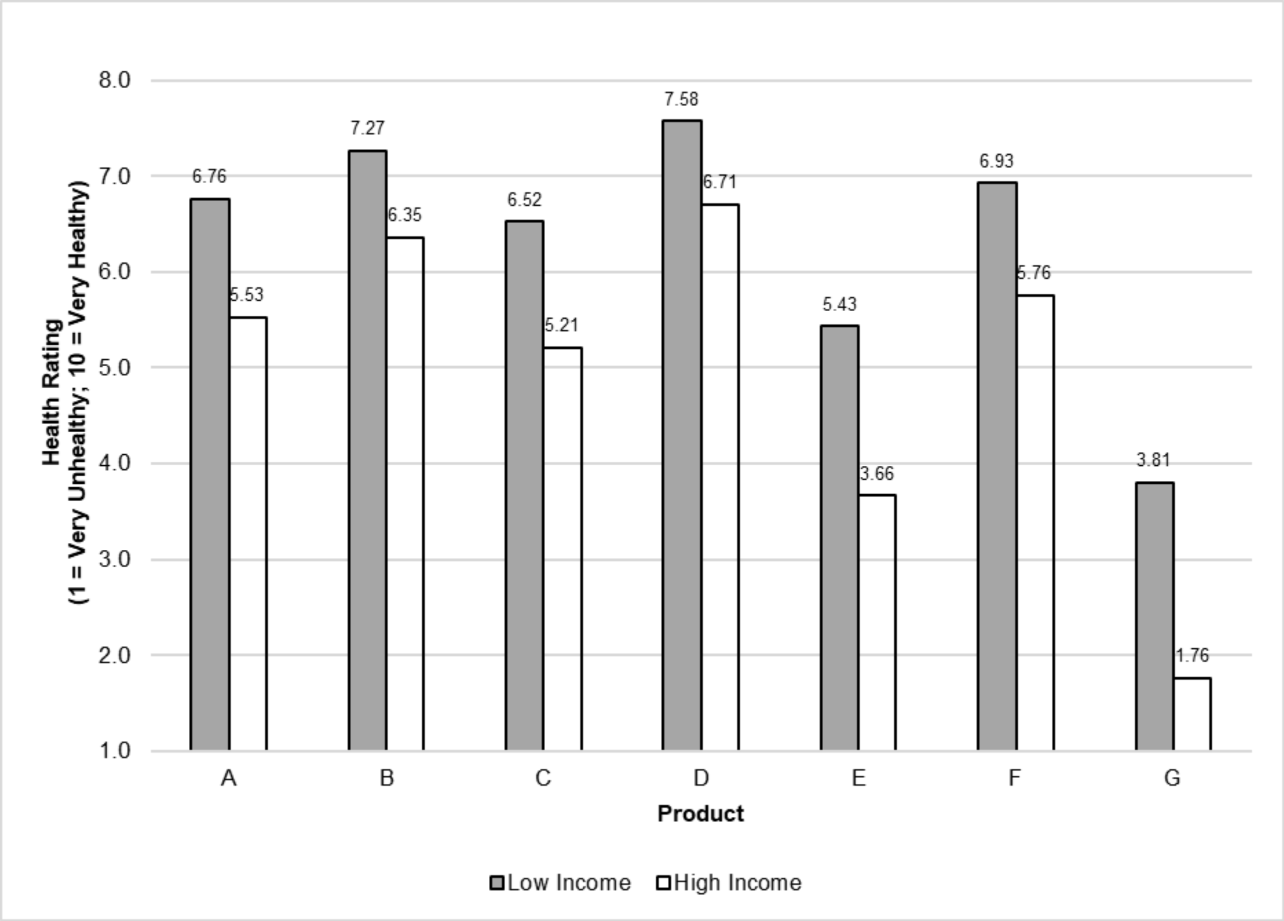

Figure S2. Income effect on label ratings for fictitious cereal product with different FOP labels. *Product A: control; Product B: high Health Star Rating; Product C: Guideline Daily Amount with a 'less healthy' nutritional profile; Product D: endorsement logo/low Glycemic Index claim; Product E: low Health Star Rating; Product F: Guideline Daily Amount with a 'healthy' nutritional profile; Product G: warning. In terms of income, low-income levels of ZAR40 000/month and high-income levels of ZAR115 000/month were used to generate a graph for illustration purposes.*

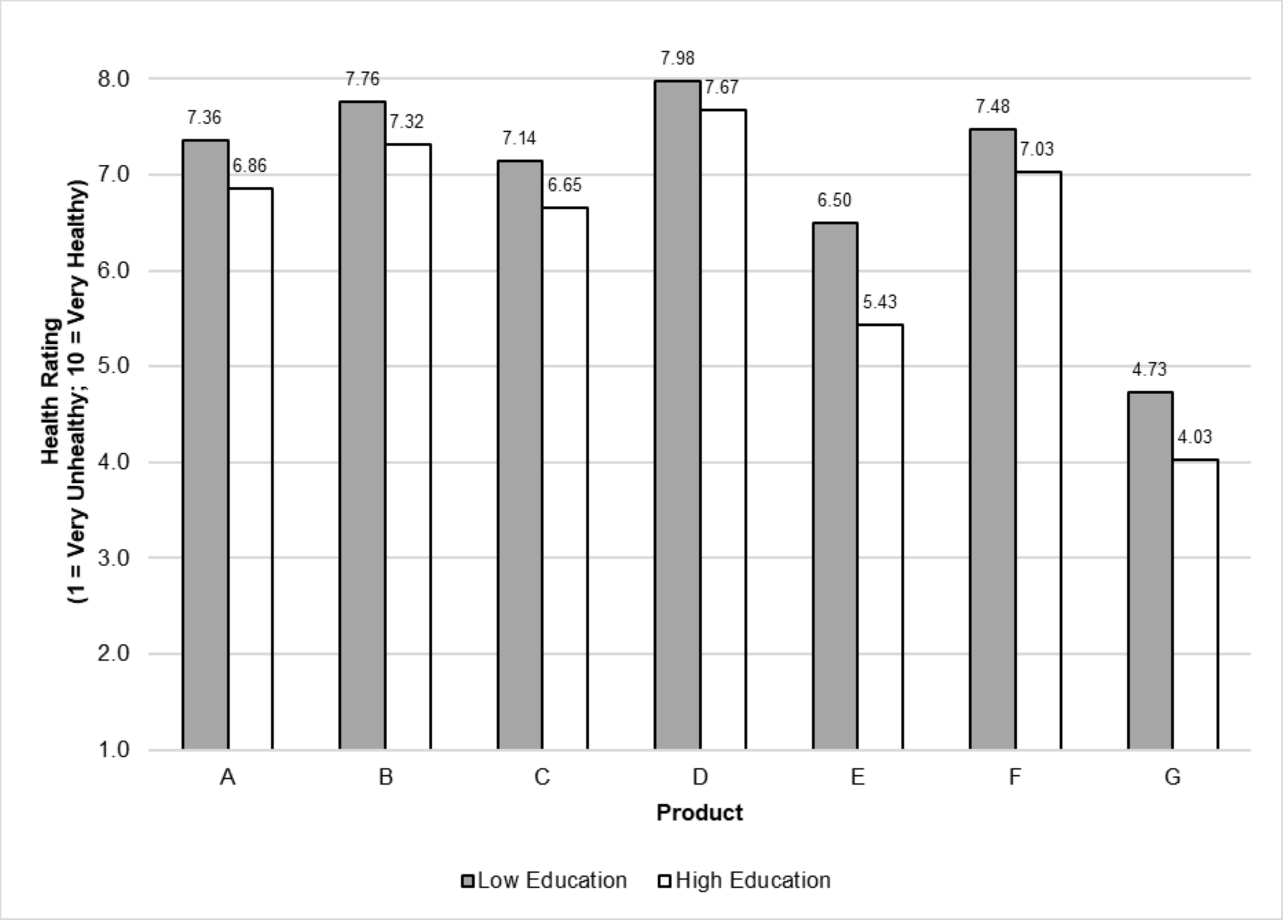

Figure S3. Education effect on label ratings for fictitious cereal product with different FOP labels. *Product A: control; Product B: high Health Star Rating; Product C: Guideline Daily Amount with a 'less healthy' nutritional profile; Product D: endorsement logo/low Glycemic Index claim; Product E: low Health Star Rating; Product F: Guideline Daily Amount with a 'healthy' nutritional profile; Product G: warning.*
